# Leveraging public health infrastructures to support sexual health for women living in the Southern United States

**DOI:** 10.3389/fmed.2025.1521455

**Published:** 2025-09-02

**Authors:** Madeline C. Pratt, Tammi F. Thomas, Anthony Merriweather, Caroline Deaterly, Bernadette Johnson, Lisa Hightow-Weidman, Kathleen E. Hurwitz, Kara Bennett, Nuvan Rathnayaka, Henna Budhwani, Aadia Rana, Ariann Nassel, Latesha E. Elopre, Lynn T. Matthews

**Affiliations:** ^1^Division of Infectious Diseases, Department of Medicine, Heersink School of Medicine, University of Alabama at Birmingham, Birmingham, AL, United States; ^2^Alabama Department of Public Health, Montgomery, AL, United States; ^3^College of Nursing and Institute on Digital Health and Innovation, Florida State University, Tallahassee, FL, United States; ^4^Target RWE, Durham, NC, United States; ^5^Lister Hill Center for Public Health Policy, School of Public Health, University of Alabama at Birmingham, Birmingham, AL, United States

**Keywords:** HIV prevention, women, deep South, Alabama, cohort, STI, HIV

## Abstract

**Background:**

Camellia is a single-arm longitudinal cohort study. We engaged a Community Advisory Board to support refinement of an existing mHealth app platform for use among cis- and transwomen in Alabama, one of seven states prioritized in the federal Ending the HIV Epidemic (EHE) strategy. We partnered with the Alabama Department of Public Health to recruit from a database of women recently diagnosed with gonorrhea or syphilis, aged 18–50, across Alabama. Potential participants are recruited by telephone. A home-based HIV and sexually transmitted infection (STI) testing program allows the study to offer tailored sexual health information, testing, linkage to care and support while assessing STI and HIV incidence and associated predictors.

**Methods:**

Study participants are enrolled into the digital Camellia Cohort in which they complete home-based HIV and STI testing and online surveys every 6 months. Participants are followed for at least 24 months or until study completion (up to 42 months). Primary outcomes include predictors, mediators and moderators for HIV and STI incidence and pre-exposure prophylaxis (PrEP) use via self-report, medical record review, and dried blood spots.

**Conclusion:**

The Camellia Cohort integrates epidemiologic methods, mHealth technology, and data science to better characterize HIV transmission dynamics and engagement in the prevention care cascade among women in an EHE focus state. This study will provide critical insights into the feasibility and acceptability of a remote, light-touch cohort design, while also providing data on STI and HIV incidence, PrEP use, and key mediators and moderators influencing prevention behaviors among women with indications for PrEP in this high-priority region.

## 1 Introduction

In the Southern United States (U.S.), Black cis- and transgender women (CGW, TGW) are disproportionately affected by sexually transmitted infections (STI), including HIV. While comprising only 13% of the U.S. population, Black individuals account for more than half of HIV diagnoses (1). Black CGW comprise 57% of new HIV diagnoses among women, and HIV prevalence among TGW is 14% (1), with nearly half of newly diagnosed TGW being Black (1). These health inequities are more pronounced in the U.S. South, where rural areas have higher HIV diagnosis rates and require tailored prevention strategies (2, 3). In Alabama, a focus state for the national Ending the HIV Epidemic initiative (4), Black CGW have eight times the HIV diagnosis rate of White CGW (5). Despite the lack of surveillance data to document HIV inequities among transgender people, these disparities are likely exacerbated among TGW who face targeted restrictive legislation in the state (5). While bacterial STIs like gonorrhea and syphilis are HIV predictors among women, a deeper understanding is needed to address HIV and STI disparities among Black cis- and transgender women in the Deep South (6).

In Alabama, an estimated 16% of the 16,626 people with HIV (PWH) remain undiagnosed (7, 8). The Centers for Disease Control and Prevention (CDC) reports that only 38% of Alabamians had ever been tested for HIV in 2023, with rural areas showing significant testing gaps (9). In formative work, our team found low testing rates in areas with high HIV incidence and prevalence (10). Although HIV pre-exposure prophylaxis (PrEP) effectively reduces HIV risk, it is underused by Black individuals and women in Alabama and the South (4, 11–14). Understanding predictors of PrEP use in these groups is essential to addressing gaps in sexual health service utilization.

HealthMpowerment (HMP) is a theory-based, multi-feature mobile health (mHealth) intervention designed to educate individuals on sexual health, promote health and wellness, and build community among young gay and bisexual men who have sex with men (GBMSM) and TGW (15–17). HMP has shown strong retention among sexual and gender minority populations, with over 80% completing 3-month assessments, supported by built-in engagement strategies (15, 18–20). The user-driven, responsive platform includes a content management system, administrative dashboard, multimedia resources, social support features, automated notifications, interactive activities, and a rewards system, accessible on iOS and Android (15, 18–20). Given the app’s prior adaptation for women in South Africa and its scalability, we selected it to support development of our light-touch cohort in the U.S. South. We adapted the HMP platform to engage and retain Alabama cis- and transwomen (hereafter referred to as women) in a fully remote digital cohort, leveraging its existing capabilities to support participation without in-person visits while systematically assessing multi-level predictors of HIV and STI diagnoses.

Importantly, this longitudinal cohort study, called Camellia Cohort, collaborates closely with public health officials to precisely identify eligible women. The Alabama Department of Public Health (ADPH) Division of Sexually Transmitted Diseases (STD) initiated the “At-Home STD/HIV Specimen Collection Initiative” in May of 2021 to reach rural populations within Alabama vulnerable to acquiring HIV and other STIs. Testing kits are mailed to clients, free-of-charge, and provide up to three-site nucleic acid amplification STI testing (gonorrhea, chlamydia, and trichomonas), syphilis antibody dried blood spots (DBS), and 4th generation Antigen/Antibody DBS HIV testing. In addition, the program provides kits for PrEP patients, which add testing for pregnancy and creatinine. Through a partnership with Imaware, clients order and receive test results via a secure portal. In the first year of the program, 3,588 kits were ordered, 45% of which were returned for processing, and all Alabama counties were reached. Tests revealed a 3.8% (*N* = 136) chlamydia positivity rate, 2.5% (*N* = 90) gonorrhea positivity rate, 16 (0.45%) new diagnoses of syphilis and 3 new cases (0.01%) of HIV (21,). Through Camellia Cohort, this ADPH initiative is augmented to broaden reach in areas with high HIV incidence but poor healthcare access.

Camellia Cohort combines epidemiologic methods, public health partnerships, mHealth technology, and data science approaches to better understand HIV transmission and prevention care cascades for women in the South. The current manuscript outlines the protocol for designing and refining the HMP app platform for Southern women, as well as the protocols for sampling, recruiting, and retaining this cohort of women recently diagnosed with a curable STI (gonorrhea or syphilis). We leverage longstanding community partnerships between public health, academic research, and reporting systems to offer free sexual health information, testing, and linkage to care.

## 2 Methods and analysis

### 2.1 Study design

**FIGURE 1:**
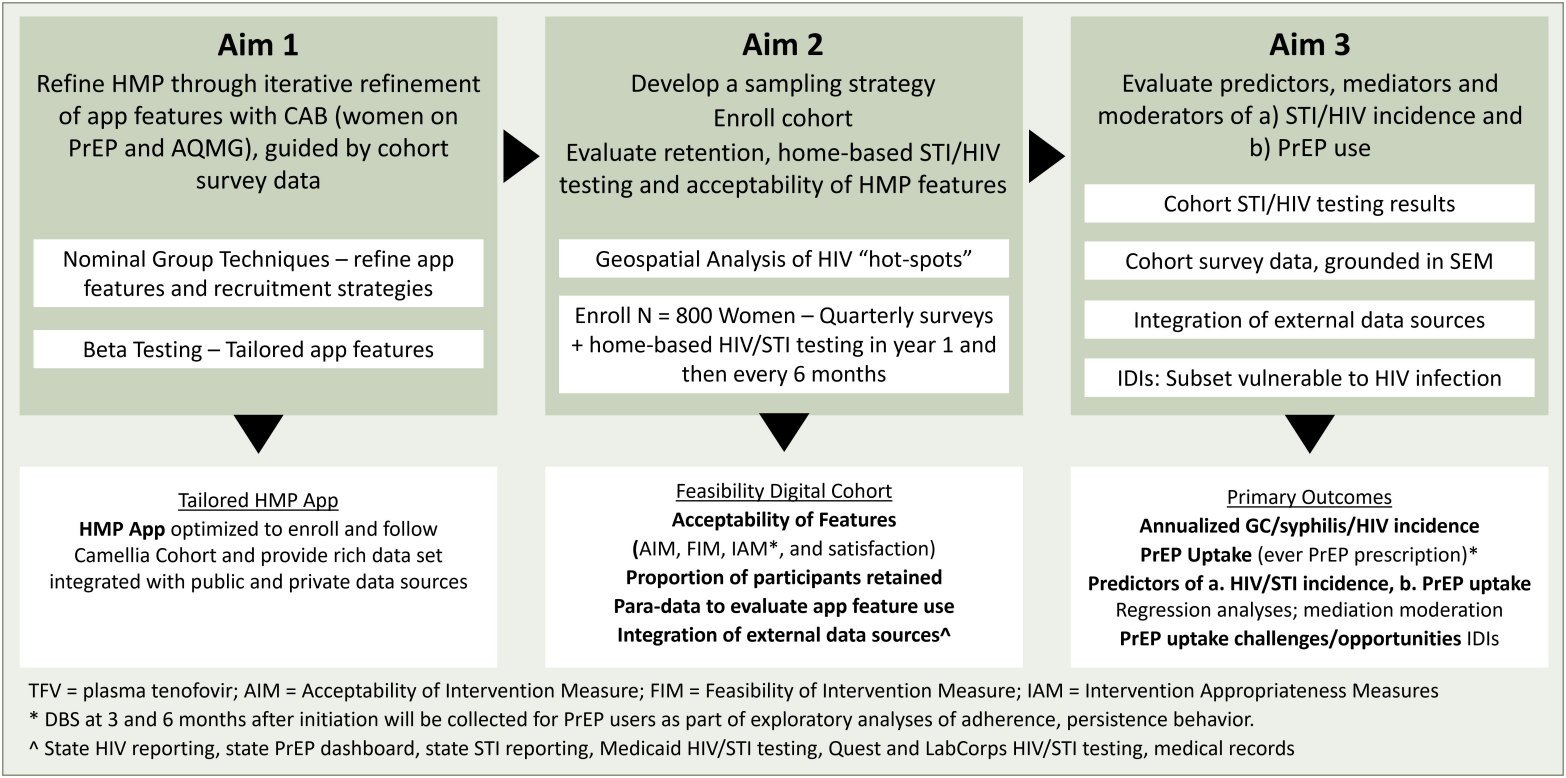
Study Overview. Aims, goals, procedures, and outcomes.

The Camellia Cohort Study is a single-arm longitudinal cohort study. A representative sample of women recently diagnosed with gonorrhea or syphilis, aged 18–50 across the state of Alabama are recruited telephonically. Study participants are enrolled into the digital Camellia Cohort in which they complete home-based HIV and STI testing and online surveys. Enrolled women are followed for at least 24 months or until study completion (up to 42 months). Primary outcomes include predictors, mediators and moderators for HIV and STI incidence and PrEP use (self-report, medical record review, and DBS) (Figure 1).

### 2.2 Conceptual framework

**FIGURE 2:**
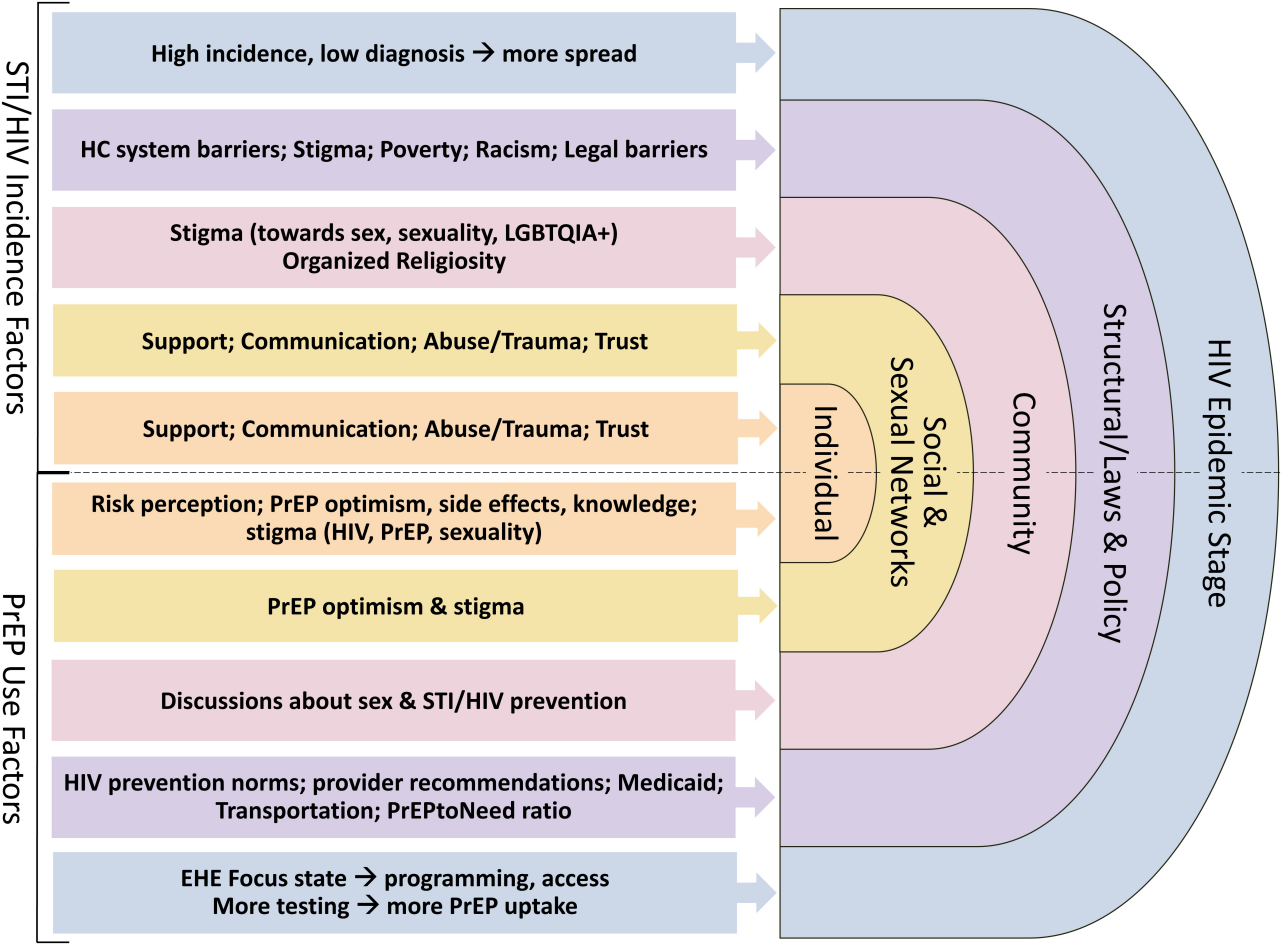
Modified Social Ecological Model (MSEM) (22) and example factors at each level that may influence HIV/STI incidence among women in Alabama (primary outcome of interest), and PrEP uptake (exploratory outcomes) based on the literature and our formative work.

We utilize a Modified Social Ecological Model (MSEM) to organize the associations between social and structural factors, individual practices, the physical environment, and health. The MSEM builds upon traditional social ecological models through its explicit focus on HIV (22) and highlights the multi-level risks and risk contexts for HIV infection; situates individual behavior within broader social networks, community, and public policy; and accounts for the epidemic stage. We adapted this model to consider multi-level influences on HIV and STI incidence and PrEP use (Figure 2).

### 2.3 Population

Women aged 18–50 who are vulnerable to HIV acquisition, based on diagnosis of syphilis or gonorrhea in the prior 3 months and their community HIV prevalence and testing coverage, are identified as potential study participants through partnership with ADPH using their STI surveillance database. This method of recruitment ensures enrollment of a cohort with demographics reflective of current inequities in STI rates across the state.

### 2.4 Study aims and outcomes

The primary aims of the study are to: (1) refine the HMP platform to include key elements that optimally (2) recruit, engage, and retain a geographically diverse and rurally enhanced cohort of women vulnerable to HIV acquisition in Alabama; and (3) evaluate predictors, mediators and moderators for HIV and STI incidence and PrEP use (self-report, medical record review, and DBS). Outcomes from the study will include the tailored Camellia Cohort digital platform and recruitment strategies, quantitative data on implementation measures (acceptability, feasibility, appropriateness, satisfaction), descriptive data of participants who were retained for the full follow-up period and completed STI testing at the requested intervals, HMP app paradata (e.g., number of clicks on a particular article, number of times a participant opens the app, amount of time spent on the app) to evaluate use of the refined app’s features, and integration of multiple data sources, including state HIV and STI reporting and commercial datasets.

### 2.5 Community advisory board

We recruited 12 community advisory board (CAB) members including C&TGW across the state. CAB members were referred by Alabama Ryan White clinics, a local organization that supports PrEP care, and by word-of-mouth and existing community partnerships. CAB members were purposively recruited to ensure at least half were from rural counties and half self-identified as Black or African American. The main goals of the CAB were to refine HMP features to optimally engage women, develop recruitment strategies, and beta-test the tailored app and elements of the protocol.

Nominal group techniques (23) were used to refine each feature within the app and to develop recruitment strategies. The CAB was given clear expectations for their role as “liaisons” between researchers and the communities, rules of conduct and general information about HIV and prevention tools. Finally, the CAB was charged to consider roles within the group and a mission statement related to improving sexual health in the South to promote participation.

### 2.6 Sampling, recruitment, and enrollment

#### 2.6.1 Inclusion and exclusion criteria

**FIGURE 3:**
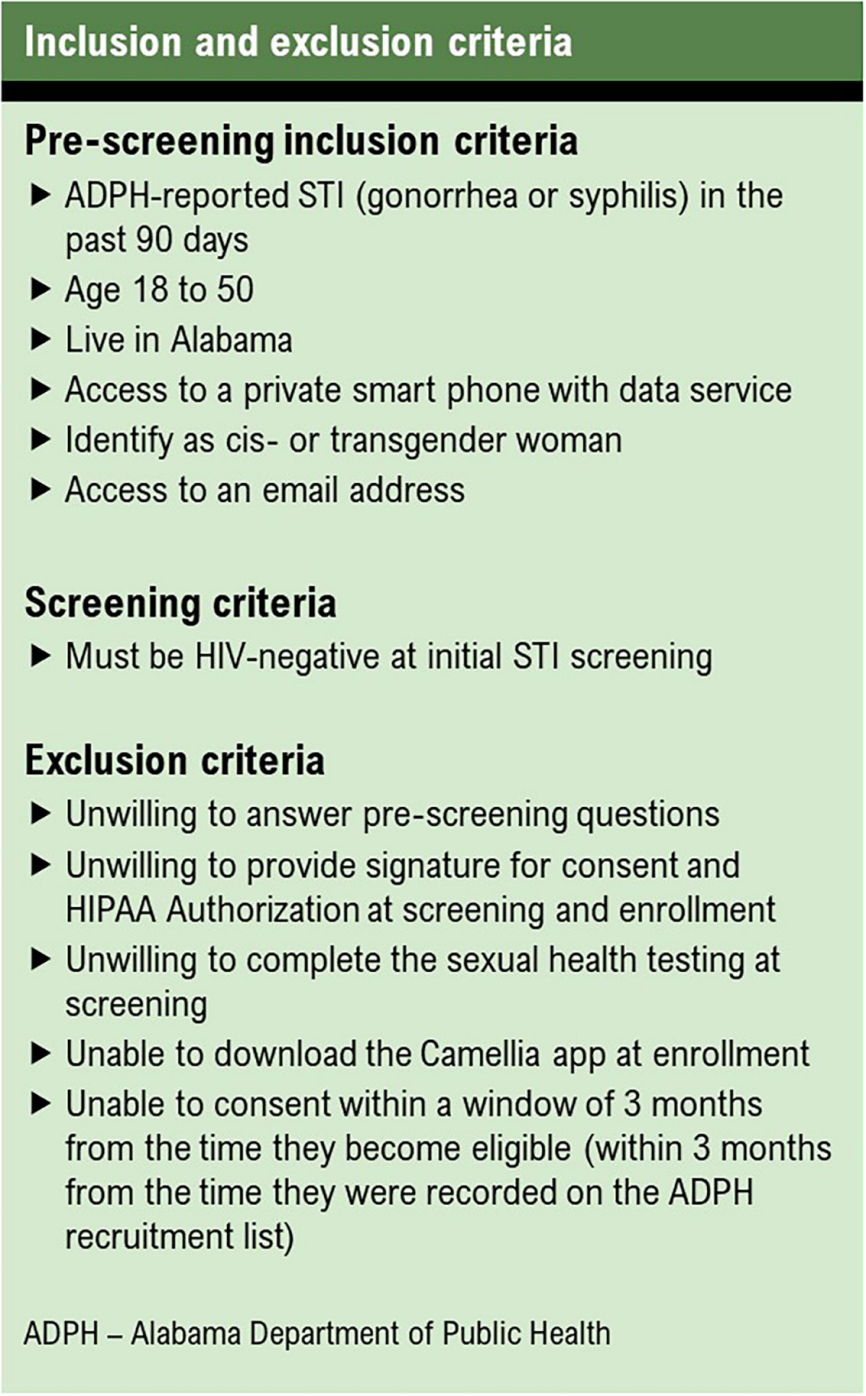
Inclusion and exclusion criteria.

To be eligible for enrollment into the cohort, participants must identify as a woman, live in Alabama, be between the ages of 18 and 50, have access to a smartphone, and have access to an email address. Women who are unwilling or unable to complete STI screening, download a smartphone app, or consent to participating are excluded (Figure 3).

#### 2.6.2 Sampling

We are using a stratified sampling framework with proportionate allocation to probabilistically identify and recruit a geographically diverse and rurally enhanced cohort of women. The sampling framework was developed based on historical HIV incidence data (available at county level), commercial HIV testing data (available at zip code level), rurality (measured using the 2010 United States Department of Agriculture’s [USDA] Rural-Urban Commuting Area [RUCA] codes (24); available at zip code level), and race/ethnicity. First, we allocate cohort recruitment slots for each of Alabama’s eight public health districts (PHD) proportional to the HIV incidence rate per 100,000 population in 2019 (5) such that PHDs with higher incidence rates have larger representation in the cohort. Next, we obtain a complete list of women aged 18–50 years with a recent (past 90 days) diagnosis of gonorrhea or syphilis from ADPH. The location information (i.e., zip code and county) provided by ADPH is used to assign zip code tabulation areas (ZCTA) to each woman. ZCTAs are advantageous in that they are real representations of postal service zip codes but maintain clear administrative boundaries and can be linked to U.S. Census data. We categorize each ZCTA according to HIV testing utilization (low, medium, high based on tertiles of the distribution with each PHD) and rurality (metropolitan, micropolitan, small town, rural; based on RUCA codes). Within each PHD, we randomly order the list of potentially eligible women provided by ADPH with probability proportional to HIV testing utilization and rurality categories such that women residing in rural ZCTAs and ZCTAs with low testing utilization being preferentially sampled.

#### 2.6.3 Prescreening

Women selected by the sampling strategy are contacted by staff via phone to explore their interest in participating in Camellia Cohort. Research staff read a prescreening recruitment script and document all call attempts with the unique participant ID. Women who confirm interest in participating are asked prescreening questions to determine eligibility. Eligible women are asked to complete a screening visit at that time or schedule the screening visit for a later date. Women with disconnected phone numbers or whom researchers are unable to reach on the third call attempt are excluded from further contact.

If a potential participant contacts research staff directly by phone, their eligibility is verified in the data provided by ADPH. If they are listed within the STI surveillance data, they are eligible to participate in a screening visit and contacted again by study staff.

#### 2.6.4 Screening

##### 2.6.4.1 Informed consent/HIPAA

Women are asked to provide an email address to receive the screening informed consent and HIPAA Authorization Form, which are able to be signed electronically via REDCap. Enrolled participants are asked to complete a medical release form to enable research staff to verify PrEP prescriptions and follow HIV/STI testing results in databases. Staff verify that the consent has been accurately completed and proceed to the next study activity.

##### 2.6.4.2 Ordering the Imaware sexual health screening kit

Study staff guide participants through creating an account with the Imaware system, including creating log-in credentials to order the screening kits. Staff inform the participant that the kit will be mailed to their home within 2 days and must be completed and returned within 14 days for eligibility. Participants complete the kit at home and use the postage-paid packaged envelope to return to a UPS drop box. Staff assist women with transportation barriers or those in rural Alabama with limited access to drop box by scheduling a UPS pick up to their home.

Participants and study staff receive their STI results using their unique log-in credentials. For positive HIV and/or STIs results, the Imaware Health clinicians and ADPH intervention team follow up with the participant for treatment linkage. If participants test positive for HIV, they are not eligible for enrollment. All test results are documented in REDCap.

#### 2.6.5 Enrollment

##### 2.6.5.1 HMP app orientation

Research staff guide participants through installing the HMP app on their phone, creating an account, and orient the participant to app features.

##### 2.6.5.2 Survey completion

Participants receive in-app notifications to complete the enrollment. The survey link is housed on the milestone tab in the app. The notification includes a unique link to complete the survey via REDCap. Participants also receive an SMS and email notification with the link to the survey.

### 2.7 Study activities

#### 2.7.1 Surveys

Following enrollment, participants complete 6-month (at the 6, 12, 18, 24, 36, 42-month time points) surveys and sexual health tests (Table 1). Reminders occur via email and HMP app notifications on day 0 (i.e., beginning of window for each visit), 3, 5, 7, 10, and 15 and include a unique clickable link to complete the survey and request a sexual health kit via REDCap. An incentive of $80 is paid upon completion of the brief survey, $110 for the annual survey, and $75 once test results are made available to staff. Over the course of the study, the total available compensation is $1,460 across the 42 months, with yearly compensation up to $340 if all study activities are completed.

**TABLE 1:** Questionnaire items - collected at enrollment and annually.

MSEM domains	Topic
Individual	Demographics
	**# pregnancies**, **# children**, **vital status of children**, **pregnancy status**
	Discrimination experiences
	Alcohol use (37, 38)
	Substance use (39)
	STI-related stigma (40)
	STI (41) and HIV (42) knowledge
	Psychological symptoms (43, 44)
	**HIV risk perception** (30)
	**PrEP knowledge and intention** (30, 31)
	Items limited to PrEP initiators: **PrEP use** (45, 46), **PrEP use stigma** (47, 48), **PrEP self-efficacy** (49), **medication side effects** (50)
	Resilience (51)
	Food insecurity (52)
Social networks	**Sexual behavior** (53–55)
	Relationship power (56), gender-based violence (57)
	Social support (58, 59)
Community	Community level HIV stigma (60)
	Sense of community (61, 62)
	Gender norms (63, 64)
Structural	Health care utilization (65)
	Medical trust (66)

Brief surveys (bolded underlined items) collected every 6 months.

The value of the incentives was chosen to encourage retention in the digital, hands-off cohort and to promote return of the at-home STI testing kits and completion of longer surveys. Additionally, participants are required to have a smartphone with internet access or a data plan, so the incentives were valued to provide support for these associated costs (Table 2).

**TABLE 2:** Camellia cohort client procedures.

Years from enrollment	1	1	1	2+	2+^@^
**Months from enrollment**	**0**	**6**	**12**	**18**	**24**
Full questionnaire	X		X		X
Brief questionnaire		X		X	
HIV testing	X	X	X	X	X
STI testing	X	X	X	X	X
In-depth interviews (*N*∼30)^#^			X		
***Additional procedures for women who initiate PrEP***					
DBS (home collection) at 3M and 6M after start^∧^		X	X		
Medical record review to evaluate PrEP rx data^∧^					

#IDIs w/N∼10 ever-PrEP users and *N*∼20 women vulnerable to HIV acquisition based on Aim 3 analyses and never accessed PrEP. ^∧^Boxes here represent schedule for a ppt who reports PrEP start (confirmed via external data) at M3. @After 12 months of follow-up, women are asked to complete annual surveys + 6-monthly HIV/STI testing until cohort conclusion.

#### 2.7.2 Laboratory testing

Participants complete the initial self-collection kit at the screening visit, and if eligible, repeat the self-collected kits at the follow-up visits. Participants request kits (single vs. triple site) based on their sexual exposure and preference. When participants first receive the kit, they activate it by scanning the QR code or visiting the website provided on the box. Then, they follow the instructions to collect their specimen and mail their completed collection kit back to Imaware within 14 days. At 7 and 14 days following the date that the kit was requested, Imaware sends an automated reminder email to return the kit. Participants receive daily email reminders once the kit has been activated.

Staff retrieve test results from the Camellia Imaware portal and enter the results into REDCap. Participants with positive test results are notified by ADPH and Imaware and cared for through the standard of care that ADPH provides. Participants found to have HIV are withdrawn from the study but continue to have access to the educational resources in the app until the study ends.

##### 2.7.2.1 HIV testing

Human immunodeficiency virus (HIV) testing is conducted using a Combo Antigen/Antibody ELISA for the qualitative detection of HIV p24 antigen and antibodies to HIV types 1 and 2 using the GS Combo Ag/Ab assay from Bio-Rad. If reactive, the result is confirmed by processing the sample in duplicate using the same assay, as well as reflexing to the Bio-Rad Geenius platform for genotyping. The assay is FDA approved with sensitivity of 100%, specificity > 99%.

##### 2.7.2.2 STI testing

*Neisseria gonorrhoeae*, *Chlamydia trachomatis*, and *Trichomonas vaginalis* are tested via PCR testing of a self-collected vaginal swab through the Imaware platform (25, 26). Sites (e.g., vaginal, rectal, pharyngeal) for testing are modified based on sexual practice profile.

##### 2.7.2.3 Syphilis testing

Dried blood spots procedures (described below) test for *T. pallidum* antibodies (IgG). This is not collected from those with prior syphilis infection as the test cannot distinguish active and prior cases.

##### 2.7.2.4 PrEP use (DBS)

Participants that report recent use of oral PrEP on the survey or in the app are asked to collect a dried blood spot (DBS) to test for medication use and adherence (tenofovir diphosphate [TFV-dp] quantified in red blood cells to determine doses per week). This self-administered test is mailed to their home. Participants must return their dried samples by mail to OraSure within 30 days. As a function of the signed HIPAA, staff request their test results. Participants complete another DBS test 3 months later in the same manner. Results for the DBS are recorded in REDCap.

#### 2.7.3 Individual in-depth interviews (IDIs)

Up to 30 IDIs will be conducted with a sample of enrolled women, including at least ten who initiated PrEP while enrolled in the cohort. Eligible enrolled women who consented to future contact are recruited via HMP app messaging, and women interested in participating will be contacted by a study team member. The interview will take 60–90 min to complete, be audio recorded and transcribed and then analyzed using content analysis.

The interview guide is informed by the MSEM framework. The guide includes descriptive and narrative questions to facilitate a more granular understanding of mediators and moderators of HIV and STI acquisition risk and prevention strategy use. Question domains include individual, community, network, epidemic, and laws and policy, specifically asking questions about making decisions about sexual health, community impact on sexual health, experiences within the study and using home-based STI testing, knowledge of and attitudes toward PrEP, and experiences with sexual and reproductive healthcare in Alabama.

### 2.8 Data analysis

#### 2.8.1 HIV and STI incidence

We use Cox proportional hazards models to obtain incidence rate ratios and 95% Wald confidence intervals to examine associations between individual- and community-level sociodemographic, clinical, and other factors, including time to first STI or HIV. Participants who are lost to follow-up (no contact after 6 months from last scheduled contact), including due to moving out of Alabama (and therefore out of ADPH catchment), are censored on the date of last contact.

To address potential confounding by measured covariates, we will adopt a propensity score weighting approach to create comparison groups of women who have similar characteristics according to the particular predictor of interest. Specifically, each woman in our cohort will be weighted by the inverse of the probability of having the predictor of interest, conditional on a set of measured confounders (i.e., propensity score). In the resulting weighted sample, the exposure-outcome relationship is preserved, but the probability of exposure is made independent of the measured confounders. A crude analysis can then be conducted in the weighted cohort (under the assumption of no unmeasured confounding) to obtain an unbiased estimate of the effect of exposure on the outcome.

We will assess covariate balance between women with and without the predictor of interest after applying the weights. If differences between the two groups remain, we will re-estimate the model used to obtain the propensity scores to allow for possible non-linearities (e.g., inclusion of higher-order and interaction terms) (27, 28). Although propensity score methods are most commonly used in the context of binary or categorical variables, they may be applied in the context of continuous exposures (e.g., income or level of social support). Similarly, recent methodological developments have extended their application to settings with multilevel or clustered data (29).

All analyses will account for the cohort’s sampling framework. We will calculate a sampling weight for each participant that reflects the unequal probabilities of selection introduced by (1) proportionate allocation at the district level and (2) preferential sampling of women from ZCTAs within PHDs according to rurality and HIV testing utilization. We will record the total number of eligible women over successive rounds of sampling to construct a sampling weight for each participant equal to the inverse of the probability of selection. The final sampling weight will be adjusted for non-response as appropriate.

#### 2.8.2 PrEP uptake

We calculate incidence of PrEP initiation–as reported by participants and confirmed through medical record review or through DBS-confirmed presence of TFV-dp–using the same approach outlined above for HIV/STI incidence. However, for the purposes of examining predictors of initiation, we only consider the first reported use of PrEP as the primary endpoint. Similar to HIV/STI incidence approach described above, participants lost to follow-up will be censored on the date of last contact. Due to anticipated lower PrEP uptake, this will be an exploratory analysis which will also be examined through qualitative methods. Intention to use PrEP (30, 31) will be assessed annually, thus yielding a repeated measures dataset. We will fit modified Poisson regression with generalized estimating equations models for this dichotomous outcome with fixed effects for follow-up time and the individual-level factor of interest along with a time by factor interaction term to assess changes in PrEP willingness and initiation over time (30). We will use generalized propensity scores to adjust for potential confounding by baseline characteristics. Predictors, mediators, and moderators will be informed by our conceptual framework. We will also conduct secondary analyses to examine app utilization by participants, including total time spent in the app and frequency of engagement with specific features (e.g., learning sessions) on stated willingness to use PrEP.

#### 2.8.3 Quantitative evaluation of mediators and moderators

We will adopt the “product method” to assess the extent to which neighborhood characteristics (e.g., rurality, poverty) mediate associations between individual-level factors (e.g., gender-based violence, substance use, depression) and HIV/STI incidence (32). For each potential mediating relationship, we will fit two regression models: (Model 1) linear or negative binomial regression (as appropriate) with the mediator of interest (neighborhood characteristic) as the dependent variable and the individual-level factor under study as an independent variable; (Model 2) accelerated failure time with the endpoint as the dependent variable and the exposure (i.e., individual-level factor) and mediator of interest as independent variables. Both models will include adjustment for all measured confounders of the exposure-mediator relationship (Model 1) and the outcome-mediator relationship (Models 1 and 2). All exposure-mediator interactions will be evaluated and included in Model 2 as appropriate. All exposure-mediator interactions will be evaluated and included in Model 2 as appropriate. The mediated effect of the individual-level predictor is obtained by combining the beta coefficients for the factor (Model 1) and mediator of interest (Model 2) (33).

We will examine neighborhood characteristics as potential moderators of associations between individual-level factors and HIV/STI incidence during cohort follow-up. We will construct interaction terms for each moderator with the individual-level factor and fit Cox proportional hazards models for each endpoint containing fixed effects for the moderator, individual-level factor, and the relevant interaction term. If the beta coefficient for the interaction term is statistically significant, this will provide evidence of effect modification.

#### 2.8.4 Qualitative data analysis

We will use a content analytic approach (34, 35)–a systematic set of inductive procedures for developing categories of information, organizing data in categories through coding, and connecting and/or framing categories for integrated interpretation. Coded data will be sorted to identify themes, and conceptual categories will be developed to describe identified themes. Data matrices will be created to visually represent relationships between key concepts and elicit patterns in the data. Throughout this process, the qualitative team will hold analysis meetings to review quotes, thematic patterns and discuss any discrepancies or disagreements that will be resolved by an additional member of the research team.

## 3 Ethics and dissemination

The protocol is approved by the Institutional Review Board at the University of Alabama at Birmingham (UAB) (Birmingham, AL, USA) (IRB-300009250) and the Research Review Committee at the Alabama Department of Public Health (Montgomery, AL, USA). The protocol is registered at ClinicalTrials.gov (NCT 05463692). A data safety monitoring board is established to ensure the safety of the data and ethical conduct of the study.

## 4 Limitations

The proposed sample size is powered to meet the primary research aims. Recognizing recruitment challenges in the rural South, we set a realistic target size, with the HMP app expected to aid engagement and retention, though ongoing support from our research team will be essential. Given limited longitudinal data for cis- and transgender women highly vulnerable to HIV in Alabama, this cohort will provide crucial insights. Through our EHE collaborations, we will incorporate diverse data sources to maximize learning from this unique group.

This is not purely observational; the HMP app actively promotes PrEP linkage and knowledge sharing. Given Alabama’s static HIV incidence since 1993 and rising STI rates, an observational approach alone is insufficient. We will explore associations between app usage frequency and key outcomes, aiming to engage and retain this population through educational content and support provided by the app.

This cohort does not specifically recruit women who use drugs; however, by recruiting women with STIs we are likely to capture women with HIV exposure through drug use and/or transactional sex (36).

Camellia Cohort does not propose to recruit women under 18 years of age. We look forward to engaging with adolescent health experts in future iterations of this work. The goal for this project is to adapt the platform and learn about HIV incidence among adult women given their highest incidence of HIV.

Including both cisgender and transgender women in this cohort may dilute insights specific to each group, especially as TGW are underrepresented in HIV studies in the Deep South. Given gender-reporting limits in ADPH STI data, our sample may have fewer TGW, but we will still adapt the cohort for them and summarize their data separately. We expect future refinements to enhance recruitment and retention of TGW in Deep South cohorts.

While we recognize that phone calls are not the most successful mode of contact for younger participant populations for recruitment, we are limited to what is approved by our IRB. We are working on ongoing adaptations to recruitment strategies that meet the standards set by our IRB while reaching our participant population as effectively as possible, including exploring opportunities for text messages as the first contact attempt.

## 5 Conclusion

This study will refine and test implementation of a mobile-based platform and enroll a digital cohort of women vulnerable to HIV in a Southern EHE-priority state with a substantial non-urban HIV epidemic. We will enhance an academic-public health-community partnership, aligned with precision public health principles focused on effectively identifying women at risk for HIV statewide in Alabama. The use of state and commercial laboratory HIV/STI datasets allows us to identify and recruit a geographically representative population of women vulnerable to HIV acquisition. This study utilizes a novel application of a modified social-ecological framework to inform multi-level evaluation of determinants of HIV risk among urban and rural women.
